# Iron supplementation of breastfed Gambian infants from 6 weeks to 6 months of age: protocol for a randomised controlled trial

**DOI:** 10.12688/wellcomeopenres.17507.1

**Published:** 2022-01-18

**Authors:** Isabella Stelle, Mamadou Bah, Sergio A. Silverio, Hans Verhoef, Ebrima Comma, Andrew M. Prentice, Sophie E. Moore, Carla Cerami

**Affiliations:** 1 Department of Women and Children's Health, King's College London, 10th Floor North Wing, St. Thomas' Hospital, Westminster Bridge Road, London, SE1 7EH, UK; 2 Nutrition and Planetary Health Theme, MRC Unit The Gambia @ the London School of Hygiene and Tropical Medicine, PO Box 273, Banjul, The Gambia; 3 Division of Human Nutrition and Health, Wageningen University, PO Box 17, 6700 AA Wageningen, The Netherlands

**Keywords:** iron deficiency, anaemia, supplements, iron, infants, global health, nutrition intervention, breastfeeding

## Abstract

**Background:** A recent analysis showed that plasma iron concentrations decline rapidly from birth in Gambian infants, irrespective of sex or birthweight, to concentrations well below normal expected values for iron-replete children older than two months of age (typically >10 μmol/L). The development and function of neural and immune cells may thus be compromised before the minimum age at which children should receive iron supplementation as per World Health Organisation recommendations.

**Methods:** This study is a two-arm, double-blind, placebo-controlled, randomised superiority trial. Infants will be randomised to receive iron drops (7.5mg/day of iron as ferrous sulphate) or placebo daily for 98 days, to test the impact on serum iron concentrations in healthy, breastfed infants (n = 100) aged 6-10 weeks at enrolment. Participants will be visited daily and supplemented by the field team. Daily health and weekly breastfeeding questionnaires will be administered. Anthropometry, and venous blood and faecal samples will be collected at enrolment and after 98 days of supplementation with serum iron as the primary endpoint. Low birthweight (less than 2.5kg at birth) and infants born prematurely (< 37 weeks) will not be excluded. Formula-fed and infants with any illness will be excluded. An additional study exploring maternal stakeholder perspectives of the intervention will be conducted by means of maternal interviews and four focus group discussions with local stakeholders.

**Discussion:** Most breast-fed Gambian infants have very low circulating iron levels by five months of age. This study will introduce iron supplements much earlier in infancy than has previously been attempted in a low-income setting with the primary aim of increasing serum iron concentration.

**Trial registration:** Clincaltrials.gov ( NCT04751994); 12 ^th^ February 2021

## Introduction

**Figure 1.**
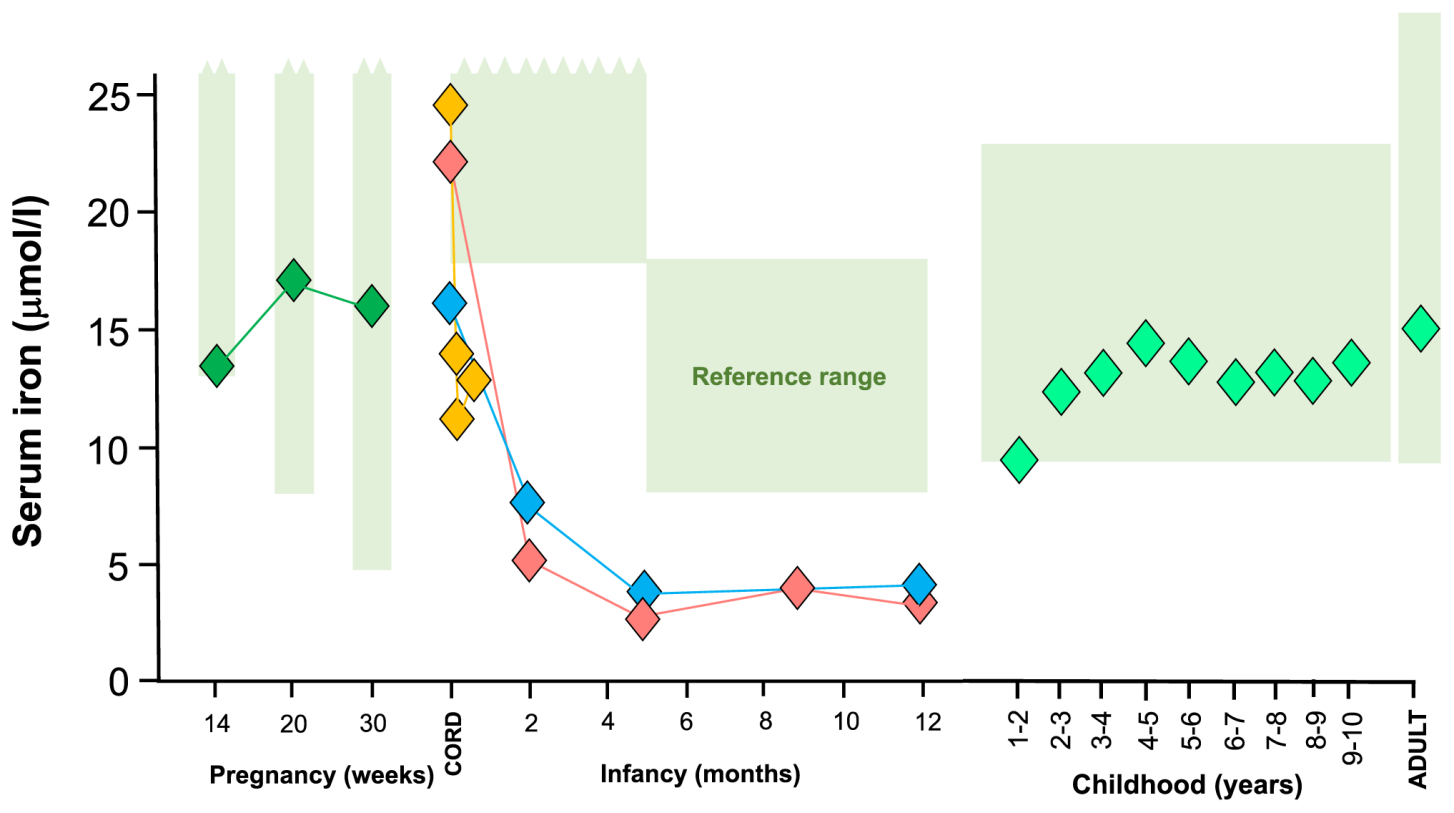
Low serum iron levels in breastfed Gambian infants and pregnant women. Data sources: Reference ranges (shaded green) ^8^. Pregnancy (dark green diamonds) ^9^. Neonates (yellow diamonds) ^10^. Infancy (blue diamonds) ^1^. Infancy (red diamonds; analysis of stored samples ^11^. Childhood and adulthood (light green diamonds); unpublished analysis from the Keneba Biobank ^12^.

In a recent study, rural Gambian infants (n = 317) had serum iron levels far below the reference range in their first year of life ^1^. The infants were born with a reasonable endowment of iron despite being born to iron deficient mothers, but following birth there was a rapid deterioration of haemoglobin and ferritin, especially in the fastest growing infants ^1^. By five months of age about 95% of the infants had low serum iron concentrations (below 5 umol/L at five months of age) and this continued beyond the first year of life. Figure 1 combines this data with other published and unpublished data from the same setting confirming that serum iron levels fall far below standard ranges for infants. Serum iron is the source of iron for growing tissues such as neuronal and immune cells ^2^.

During the first three years of life there is rapid myelination especially of the frontal cortex and basal ganglia (motor control) ^3^. Infants with iron deficiency can have symptoms that are consistent with impaired hippocampal function, reduced myelination, altered temperament and dopamine metabolism. Iron-deficient infants can present with decreased attention and memory ^4^ with deficits in visual and auditory systems as well as altered temperament, and social and emotional behaviours ^5^. A large body of evidence both from humans and animal models indicates that iron deficiency in early life can alter the brain and nervous system ^6^. Since circulating iron is the only iron source for circulating immune cells it may be anticipated that immune function, including appropriate responses to childhood vaccination, might also be compromised ^7^.

Therefore, the goal of this pilot study is to introduce iron supplements to breastfed babies much earlier in infancy in a low-resource setting with the aim of improving serum iron concentration. If this trial is successful, we plan to conduct a larger trial that includes behavioural, cognitive and immune assessments.

### Study objectives

The primary objective is to measure the impact of daily iron supplements for 98 days starting at 6–10 weeks of age on serum iron concentration at the end of the intervention in breastfed Gambian infants. The secondary objectives are to determine if iron supplementation at this age will affect: (1) the duration of exclusive breastfeeding, (2) growth, (3) pathological processes in the gut, and (4) the frequency of adverse events.

Additionally, a qualitative study will assess the acceptability of the iron supplementation efficacy trial. The supplementary objectives are to:

a) Explore mothers’ (the main care providers) perspectives on the acceptability of iron supplementation in their young, breast-fed infants through in-depth interviews.
b) Explore the acceptability of iron supplementation in young, breast-fed infants through focus group discussions with local stakeholders.
c) To integrate the quantitative and qualitative clinical intervention outcomes to explore the acceptability of iron supplementation in young, breast-fed infants.

## Protocol

### Study design and site

This is a two-arm, double-blind, placebo-controlled, individually randomised trial comparing children (n=100) who receive daily supplements with or without iron (7.5mg/day of iron as ferrous sulphate) for a duration of 98 days.

The study is based at the Medical Research Council (MRC) Unit the Gambia (MRCG) at the London School of Hygiene and Tropical Medicine (LSHTM) (MRCG@LSHTM). The study operates out of the MRCG@LSHTM Keneba field station and the Jarra Soma Regional Hospital in the Lower River Region. Study participants will be recruited from the Soma region of The Gambia through community-based clinics and birth attendants.

### Participants

***Recruitment, informed consent, and screening.*** Healthy infants from the local communities will be identified at six to ten weeks of age. The eligibility criteria will be explained to their parents/guardians, and they will be invited to join the study by providing consent for their infant. Participants must meet all the inclusion criteria and none of the exclusion criteria to be eligible to participate in the trial.

**Informed consent**

Individual written consent for the study will be sought. Field workers will be trained to explain the project in full detail to the eligible participants’ parents/guardians, covering all aspects of such study as laid out in the ‘Participant Information Sheet’ (see *Extended data*). Literate parents will be given the Participant Information Sheet whilst illiterate parents will have the full Participant Information Sheet read to them in a language they understand. Illiterate consenting subjects will require an independent literate witness. Any questions that arise will be answered by the field workers and parents/guardians will be given the possibility to obtain further clarifications and explanations by speaking to one of the study investigators. All potentially eligible infants whose parents/guardians provide consent will be invited to a baseline visit.

### Eligibility criteria

***Inclusion criteria.*** Inclusion criteria are: infants (male or female) aged between 6–10 weeks. Infants must be exclusively breastfed with plans to continue breastfeeding through six months of age. Parents/guardians (with the participant) must reside in the study site area and be able and willing to adhere to all protocol visits and procedures. Infants must be healthy with no current illness and no chronic health problems. Informed consent must be obtained from the participants parent/guardian. Low birth weight infants (less than 2.5kg at birth) or infants born prematurely (less than 37 weeks) will be included.

***Exclusion criteria.*** Exclusion criteria include formula fed infants or those whose parents/guardians are planning to use commercially available infant formula before six months of age. Infants may not have acute illness, however, once acute illness is resolved, participants may be re-revaluated for eligibility. Infants with fever (a body temperature greater than 37.5°C or mother report of fever) within three days prior to randomisation will be excluded (once fever/acute illness is resolved, participant may be re-revaluated for eligibility). Infants of parents/guardians unwilling for their infant to avoid the ingestion of supplements or herbal/other traditional medications during the study period will be excluded. Infants with any history of, or evidence for, a chronic clinically significant disorder or disease (including, but not limited to, immunodeficiency, autoimmunity, congenital abnormality, bleeding disorder, and pulmonary, cardiovascular, metabolic, neurologic, renal, or hepatic disease), self-reported history suggestive of meningitis, seizures, Guillain-Barré syndrome, or other neurological disorders, or any condition that in the opinion of the investigator may compromise the safety or well-being of the participant or compromise adherence to protocol procedures, will be excluded.

### Baseline

**Figure 2.**
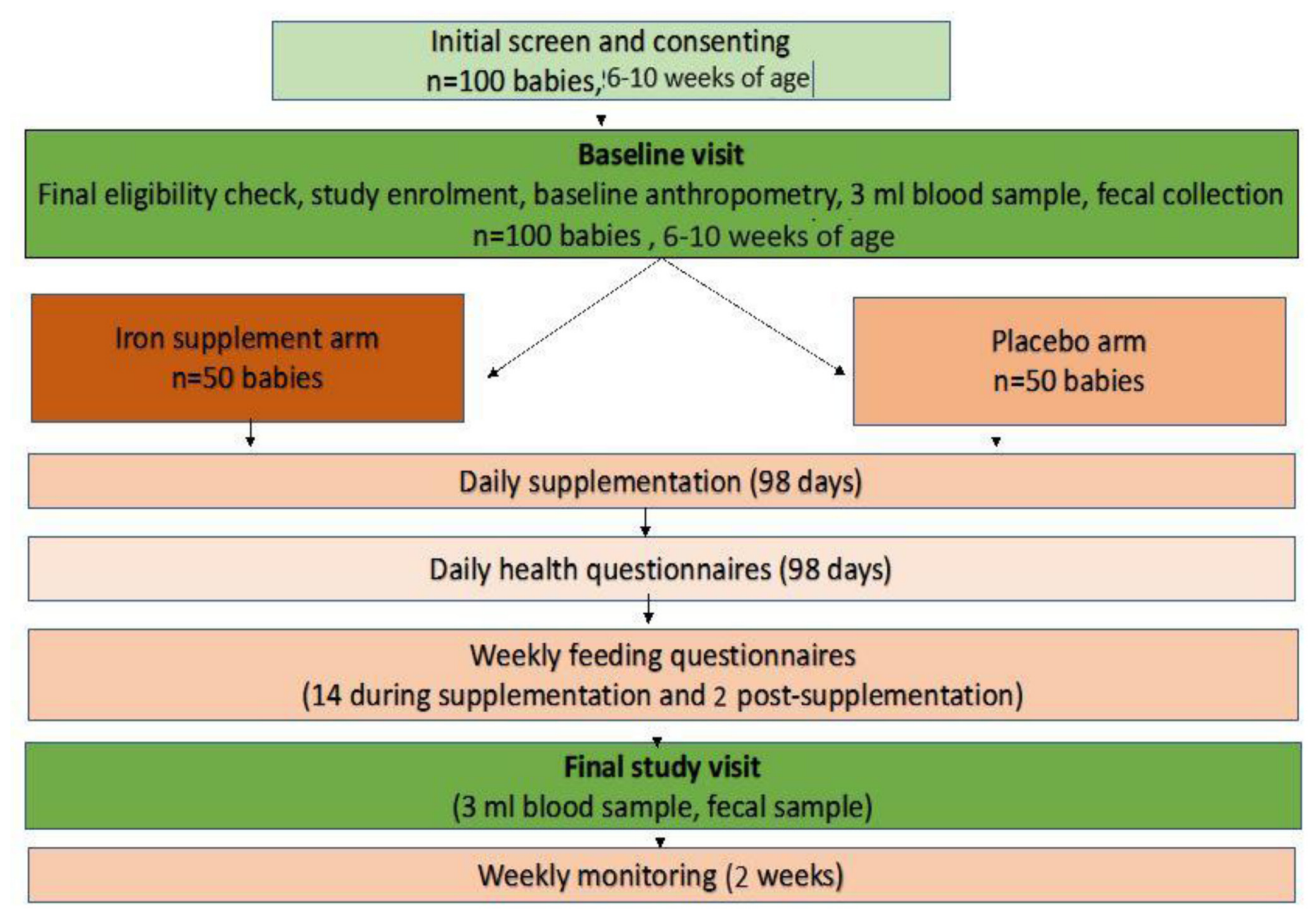
Pilot study schematic.

Once parents/guardians of the infant have signed the informed consent document, the infants will be physically examined by a study nurse and, if the infant is considered as generally healthy, their height and weight will be measured. At baseline, a 3 ml blood sample will be collected (0.5 ml in EDTA tube and 2.5 ml in serum tube) to assess the full blood count (including reticulocyte count) and to determine iron status. Parents/guardians of participants will be asked to place nappies inside-out on participants the morning before the baseline or endline visits. Faecal samples will be collected from the nappies by field workers using a spatula. This will be put in universal containers and kept in a cold box. Both blood and stool samples will be transported to the Keneba laboratory for processing and storage into -80°C within 4hrs after collection. Participants who do not pass stool will be provided with labelled universal containers and a cold box to collect faecal samples. The sample will be transported and processed by the field supervisor. This will be stored immediately into a −20°C freezer in Jarra Soma. Frozen stool sample will be transported and stored in Keneba at −80°C. Infants with haemoglobin concentration < 70 g/L will be referred to the regional health centre for treatment according to national guidelines. For safety purposes, all infant’s health will be monitored by the field workers for two weeks post supplementation. See Figure 2 for the full study scheme.

### Randomisation and blinding

Participants will be individually and randomly allocated on a 1:1 ratio to daily oral supplementation for 98 days with either iron or placebo. A randomization code will be produced in R software (see *Extended data*) and incorporated in REDCap software by the database manager (who is not part of the field team), with support from the trial statistician. The data manager, all investigators, and the entire study team are blinded to the intervention codes. The randomisation module will be protected by the RedCap “user access privilege” function to ensure that the randomization codes are not changed. Each participant will be assigned to a unique intervention code that is only known by the dedicated person independent from the study. This will prevent the unblinding of the entire study should there be a request for unblinding from the Data Safety Monitoring Board (DSMB) in the case of a serious adverse event. A list of identification numbers will be generated in advance by the MRCG@LSHTM data team with the oversight of the database manager. The study identification number (Study ID) will comprise a three-letter study code (IRN), a three-digit unique number (e.g. 001–140) and a check letter (e.g. A-Z). Every consented participant will be assigned a Study ID (e.g. IRN001X). At the end of the baseline visit, after eligibility has been fully established, the database manager/sub-investigator will allocate each Study ID to one of the six intervention codes (pre-labelled bottles of either intervention or placebo) using the randomisation module on Redcap. 

We will use permuted block randomisation with a fixed block size of six (three replications of two treatment groups within each block) to produce a list with the Study IDs. To assist in the blinding to treatment, the six numbers (1–6) will be linked to two intervention groups, so that each intervention (iron or placebo) will be replicated 3-fold within a permuted block, and arbitrarily assign the alphabetical letters to intervention groups (e.g., 1, 4, 5 indicate iron; 2, 3 and 6 indicate placebo).

A pharmacist will pre-label and re-bottle the intervention and placebo using the randomisation codes 1–6. Only the pharmacist (an independent person not involved in the trial) will know the interpretation of the randomisation codes (1–6). The codes will be put in a sealed opaque envelope and stored in a locked box. Once a participant’s eligibility to participate in the trial has been fully established and confirmed in REDCap, the participant will automatically be allocated to the next available treatment code. This treatment code with the corresponding study ID will be sent to the study nurses via email for labelling and dispensing to the field workers.

### Interventions

Supplements will be administered as drops from a liquid formulation containing either iron (7.5mg contained in 0.5mL) or its placebo (sorbitol solution USP 70%, an inactive ingredient in the iron supplements). The intervention and placebo will be issued in identical dark brown plastic bottles. The ferrous sulphate (intervention) and sorbitol (placebo) will be manufactured such that they are identical in terms of taste and appearance thus, allowing robust allocation concealment. Infants will be visited in their homes daily for 98 days by the field workers who will administer the drops. Products will be stored at ambient temperature (15–30˚C), away from direct sunlight, at both the MRCG@LSHTM Keneba Field Station main store and Jarra Soma health clinic in a locked room. The procured products have a 12-month shelf-life and expiry dates will be checked by the sub-investigator during study implementation.

### Follow up

The final study visit will be on Day 99 after 98 days of supplementation. Procedures for blood and faecal collections and storage and anthropomentry will be as per baseline. 

An early termination visit may occur because of a participant’s voluntary withdrawal, trial team decision, or at the discretion of the DSMB. Apart from the safety evaluations, no other evaluations required for the final study visit will be done.

### Laboratory evaluations

*Blood samples*: Blood samples will be taken by venipuncture into EDTA and serum tubes and kept at 2–8°C in cool boxes. Within four hours of collection the samples will be processed by the MRCG@LSHTM laboratory. EDTA samples will be run on an automated haematology analyser (Sysmex XN 1500) to measure haemoglobin concentration, to obtain a full blood count and to assess reticulocytes. Serum samples will be centrifuged and aliquots will be stored at -80°C. Samples will be analysed using a biochemistry analyser (Cobas Integra 400 plus) for iron markers (ferritin, transferrin, transferrin saturation, unbound iron-binding capacity, soluble transferrin receptor, and inflammatory markers [c-reactive protein/alpha-1 acid glycoprotein]). One of the serum aliquots will also be used to measure hepcidin, erythroferrone and erythropoiein concentrations using commercially available ELISAs.

*Faecal samples*: At baseline and endline a faecal sample will be collected, and iron content measured. Gut pathogens including bacteria, protozoa, intestinal helminths and viruses will be detected using real-time polymerase chain reaction multiplex assays. 

### Outcomes

*Primary:* Serum iron concentration on Day 99, after 98 days of iron supplementation and a one day wash-out period. Serum iron will be measured in venous blood collected at trial enrolment and 98 days after initiation of iron supplementation.

The primary endpoint is serum iron concentration at Day 99.

Secondary endpoints relating to iron status (all at Day 99) are:

a) Haemoglobin concentration.
b) Percentage of infants with anaemia (defined as: haemoglobin < 110 g/L).
c) Percentage of infants with iron deficiency anaemia (defined as: haemoglobin < 110 g/L, soluble transferrin receptor /logferritin ratio < 2.0, and ferritin < 12 ug/L or < 30 ug/L in the presence of inflammation).
d) Hepcidin concentration.
e) Reticulocyte number.
f) Erythropoietin concentration.
g) Erythroferrone concentration.

The secondary objectives are to test whether iron supplementation at this age is safe.

Endpoints related to this objective are:

a) Maternal reports of illness.
b) Adverse events.
c) Serious adverse events.
d) Changes in gut pathogens and microbiota during the study period.
e) Markers of environmental enteric dysfunction (EED).

The study will also assess potential impacts on growth, breastfeeding and feacal iron losses:

Endpoints related to these objectives are:

a) Anthropometric Z-scores relative to the World Health Organization (WHO) Growth Reference.
b) Duration of exclusive breastfeeding.
c) Faecal haemoglobin and iron.

### Mixed methods study of parent and stakeholder attitudes

The mixed methods approach will explore the effects of the clinical intervention on relevant outcomes while observing and gathering information on implementation. We will use qualitative and quantitative methods to explore the acceptability of the intervention proposed in the main study. Given that the main study is an efficacy trial, it is not possible to assess the full implementation and scale up of such a trial to the real-world setting. Instead, the proposed research will explore the acceptability of iron supplementation in young infants.

***Consent for mixed methods study.*** Prior to conducting focus group discussions and in-depth interviews, topic guides and interview schedules will be piloted among the study team. One investigator and a second female interviewer will pilot the focus group topic guides on members of the study team and adjustments will be made from the feedback. For practice, mock in-depth interviews will then be conducted by the female interviewer on the study team members. Not only will this help adjust the focus group topic guides and in-depth interviews schedules, but likewise, this will act as an opportunity for the interviewers to familiarise themselves with the questions and structure of the focus group discussions and in-depth interviews.

All potential participants for the qualitative data collection will be given written information in English in the form of a participant information leaflet and given adequate time to read it before agreeing to take part. Illiterate participants will have the information leaflet read to them by a trained member of the research team in their preferred language who will also sign to confirm their participation in consent. A copy of the signed informed consent form will be given to the participants.

### Data collection, management, and processing

Type of data: Four types of data, quantitative (demographic questionnaire and anthropometric) and qualitative (focus group discussions and in-depth interviews), will be collected.

***Quantitative.*** Data collected from questionnaires on demography, daily review of 24-hour recall of health/morbidity from parents/guardians and weekly feeding questionnaires.

Anthropometric measurements, vital signs, adverse events, and stool questionnaire.

The clinical intervention from which we will gain the quantitative materials will be from the pilot study. We will use data collected from health questionnaires and from study endpoints. This data will be collected on site and stored via a secure data capturing platform (REDCap). This data will be collected by the field workers and will be fully anonymised. The data used for quantitative analysis will include:

- Baseline demographic data
- Laboratory data
- Adherence rates
- Daily health data (adverse events and serious adverse events)
- Weekly infant feeding data

For the quantitative data, no patient-identifiable data will be included. The consent and data collection for this component will fall under the remit of the pilot study.

***Qualitative***

**Focus group discussions with local stakeholders**

Initial focus group discussions will take place with local stakeholders to further inform the in-depth interviews with infants’ mothers. Four focus group discussions will take place with:

a. Members of the National Nutrition Agency
b. MRCG@LSHTM field workers working on the study
c. MRCG@LSHTM field workers not working on the study
d. Mothers from surrounding villages whose infants are not enrolled in the study

These focus group discussions will last on average one hour and will be conducted in English or Mandinka (the predominant local language) by an investigator and a female local team member trained in qualitative research. The location will be chosen to add comfort and will most likely include the offices of National Nutrition Agency, the facilities of MRCG@LSHTM, local health clinics, and within local villages. Both MRCG@LSHTM staff from the main study and those not working for the pilot study will be interviewed to ensure a wide range of opinions are sought given that staff hiring at MRCG@LSHTM is done by application and staff applying to work on the main study may have different pre-conceived perceptions of the acceptability and necessity of iron supplementation.

**In-depth interviews with infants’ mothers**

The in-depth interviews will discuss the acceptability of iron supplementation in infancy from the perspective of mothers, but data obtained from the focus group discussions will be used to finalize the interview schedules. Using these interview schedules, 30–60-minute individual in-depth interviews will be conducted with mothers whose infants are enrolled in the trial. Interviews will be conducted, in Mandinka, by a qualitatively trained, female member of the study team, and will be audio recorded. In-depth interviews will employ concurrent and retrospective data collection by interviewing mothers during the last four weeks of the trial and in the two-week follow-up window. The location will be chosen by the interviewee to add comfort and will most likely include the homes of participants and within local villages. Data will be collected towards the end of the infant’s enrolment so that mothers have had mostly the full experience until that point.

All answers obtained and information given through both focus group discussions and in-depth interviews will be anonymised, collated, and analysed. NVivo (2020 release) software will be used to analyse qualitative data. 

### Data quality and standards

As per MRCG@LSHTM guidelines, principles of good clinical practice will be adhered to, and all training will be documented. The studies are monitored by the MRCG@LSHTM Clinical Trial Unit. To ensure standardisation of processes, MRCG@LSHTM standard operating procedures with respect to trial management, quality assurance, data management, IT security and statistics will be followed.

### Sample size

**Figure 3.**
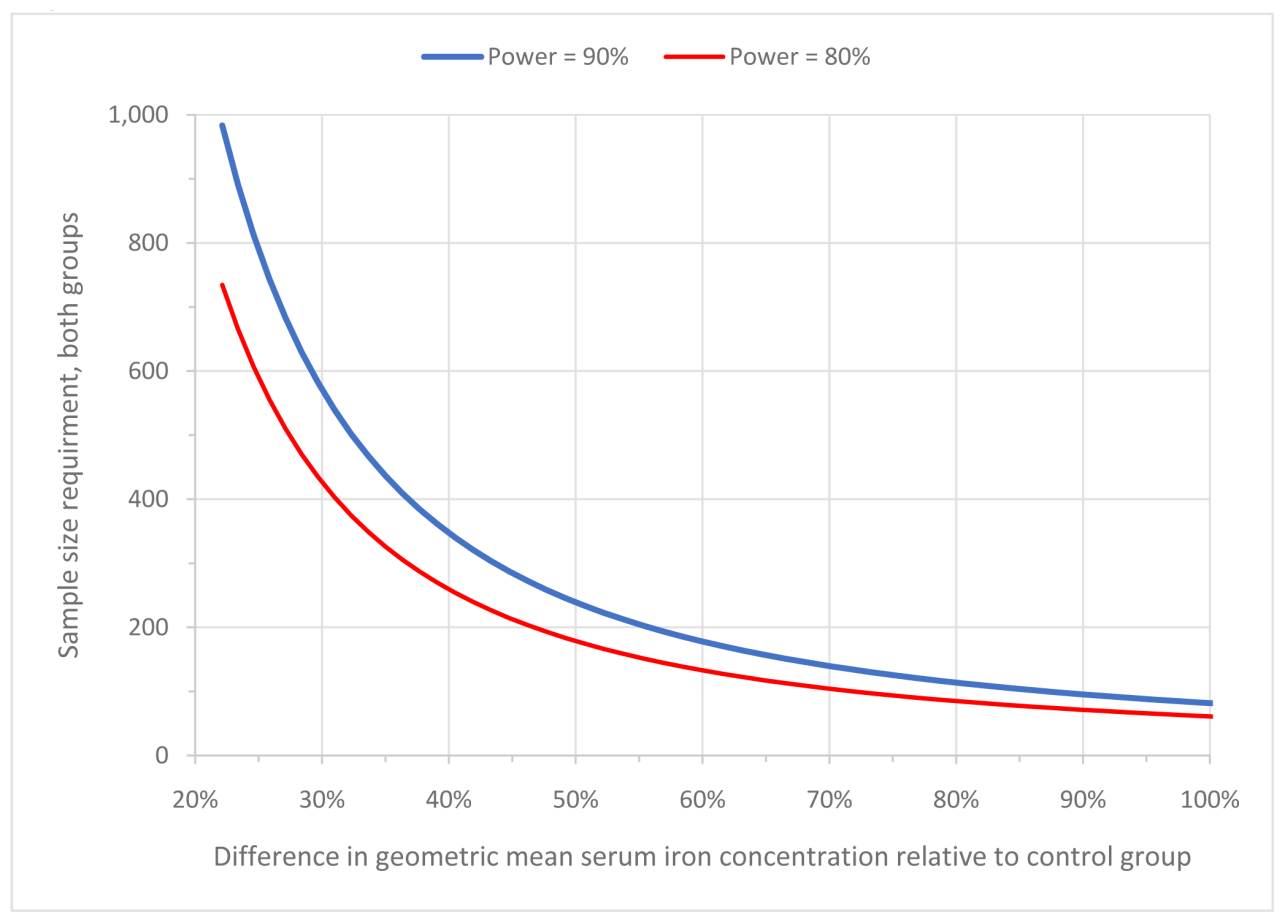
Sample size requirements for various intervention effect sizes.

We intend to enrol 100 infants in the trial, which will be sufficient to have 80% probability that, if the true increase in geometric mean serum iron concentration in the iron group increases by 70% or more relative to the control group, the 95% confidence interval for the ratio would exclude zero (with the additional assumptions that the variance of log _e_ serum iron concentration [in µmol/L] is 0.76 for each group ^13^; 10% of infants in the iron group stop taking iron supplements in the course of the intervention period; and the percentage of infants in the control group who stop taking placebo supplements is negligible) ( Figure 3). In absolute terms, an increase by 70% corresponds to a change of geometric mean serum iron concentration of 3.0µmol/L, assuming that this concentration is 4.3µmol/L in the placebo group (as observed at Day 0 in Armitage *et al.* 2019 ^1^), so that it will have increased 7.3µmol/L in the iron group. We consider intervention effects below 3.0 µmol/L (absolute group difference in geometric means) to be unimportant from medical and public health points of view.

With regards to the mixed methods study, we aim to recruit roughly five participants to each focus group discussion, in accordance with general recommendations for focus group size in qualitative research (20 participants total) ^14^. Maximum variation purposive sampling of the mothers whose infants are enrolled in the main study will be used to ensure a wide variety of the dimensions of interest, aiming to identify central themes across this diverse sampler ^15– 17^. This will take into consideration various regions, religions, families, ages, and schooling levels. However, all mothers must be Mandinka (the most common local tribe in anticipation that the majority of participants recruited into the main trial will be Mandinka) to provide homogeneity in the translation of the in-depth interviews. An equal number of mothers from the intervention and control arms of the trial will be included, to ensure both perspectives are considered. We anticipate an approximate sample size of 10–20 infants’ mothers.

### Statistical analysis

***Main study.*** Statistical analyses will be performed using R studio and STATA software. Intervention groups will be described using conventional summary statistics (e.g., means or counts with standard deviations, medians with 25- and 75-centiles). The primary analysis concerns the effect of intervention on serum iron concentration at Day 99 (i.e., 98 days of intervention with a 1-day wash-out period after administration of the last supplement, assuming that a transient increase in serum iron concentration following iron supplementation will disappear within this period), adjusted for serum iron concentration measured at baseline. Continuous outcomes will be analysed by linear regression, with transformation of the outcome as needed to normalise the distribution of residuals. Analysis will be by modified intention-to-treat, i.e., all infants who were randomised and who received at least one supplemental dose will be included. We also plan to perform per protocol analysis (i.e., to assess the efficacy that can be attained under controlled conditions) to determine the sensitivity of our modified intention-to-treat analysis. To account for missing data, we will use multiple imputations. In secondary analyses, we will adjust for any group imbalances that may occur in prognostic variables measured at baseline. Serum iron concentrations are known to undergo diurnal fluctuation and they are known to be reduced in inflammation ^18^. Thus, to account for these effects, we will also conduct secondary analyses with adjustment for the time of blood collection and for plasma concentrations of C-reactive protein and alpha-1 acid glycoprotein at the time of blood collection.

Group differences in adverse events will be analysed using negative binomial regression. In this analysis, we will count adverse events as the number of days with adverse events divided by the total number of days that the infant was observed during the intervention period. In the analysis of diarrhoea, we will count an event if the parent/guardian reported three or more fluid stools (as indicated by a WHO stool chart) in the previous 24-hour recall period ^19^. To produce conservative effect estimates, analyses of adverse events will be per protocol.

Lastly, we will explore to what extent the magnitude of the intervention effect on serum iron concentration depends on iron status at baseline, as indicated by plasma concentrations of ferritin and soluble transferrin receptor (with adjustment for plasma concentrations of c-reactive protein/alpha-1 acid glycoprotein at baseline as inflammation markers).

***Mixed methods study.*** The focus group discussions and in-depth interviews will be recorded and later transcribed and translated (where needed) into English. Translation will occur at MRCG@LSHTM by a trained employee literate in both Mandinka and English.

**Figure 4.**
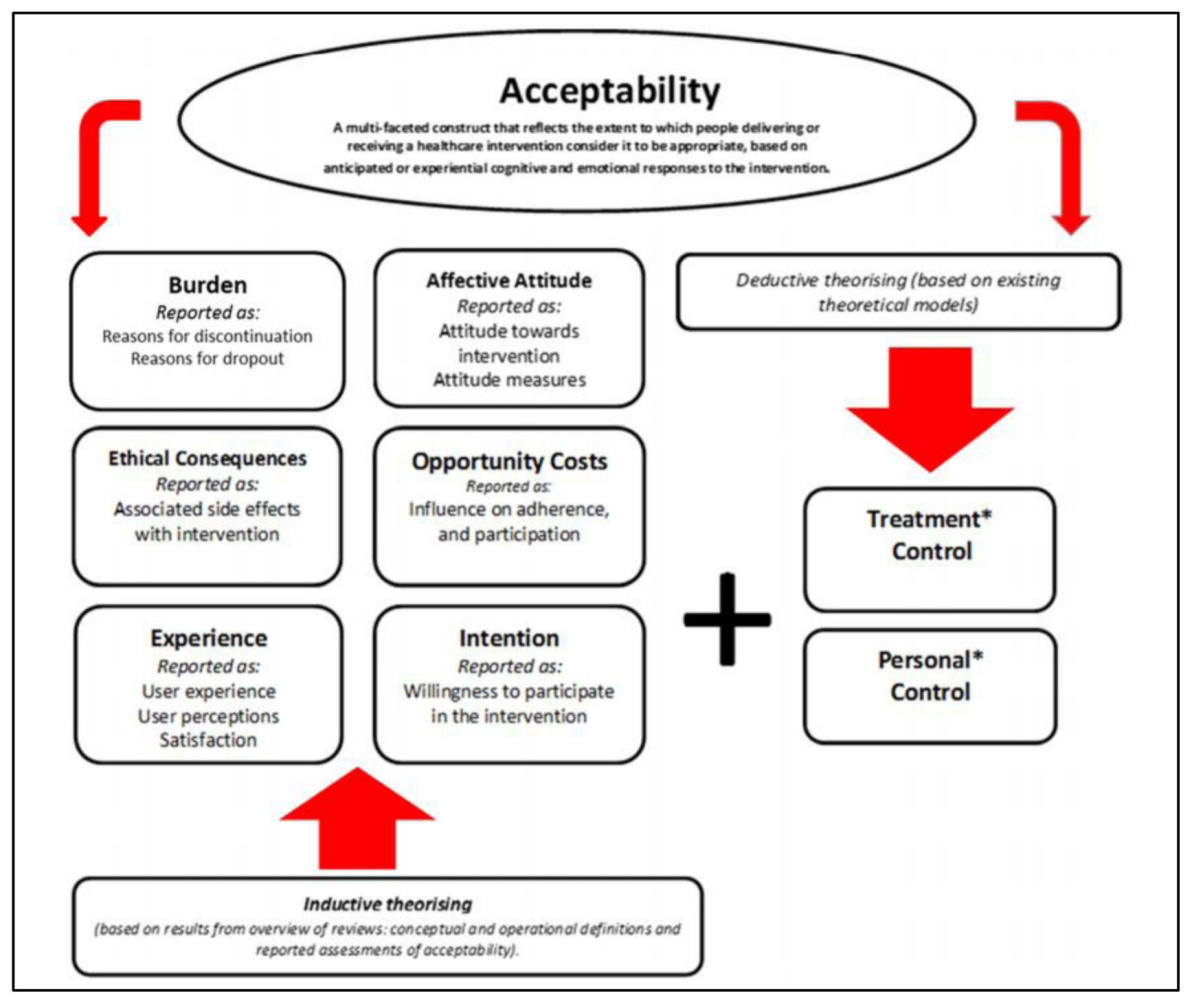
Acceptability of healthcare interventions: an overview of reviews and development of a theoretical framework (v1).

Data will be analysed with respect to the implementation framework known as *Acceptability of healthcare interventions: an overview of reviews and development of a theoretical framework* ( Figure 4) ^20^.

The qualitative data will be uploaded into, managed, and analysed in NVivo (2020 release) using a Template Analysis Method ^21, 22^. The template will be based on the six themes in the acceptability framework, with some minor variation to make analytical sense with regards to our data. By this we mean the framework itself is a macro-level conceptual framework and will require semantic changes to some terminology for it to be useful to analyse at the meso-level (i.e., data from focus group discussions with stakeholders) and at the micro-level (i.e., data from in-depth interviews with women enrolled in the trial). Template analysis follows a methodical six-stage process: 1) Data re-familiarization; 2) Preliminary coding; 3) Thematic organization within the template; 4) Defining the template; 5) Application of template to the full dataset; and 6) Finalization of template definitions. By utilising the acceptability framework as the basis for the analytical template in both the focus group discussions and in-depth interviews datasets-, the qualitative data will be able to be linked coherently across all levels. An analysis in this detail should provide valuable insight about the acceptability of the intervention. We acknowledge that the domains may not all reach saturation, but as these are based on an extensive search of the literature by the framework authors, and have been tested in different fields, we propose to use these as a basis for our analysis. We will later analyse the extent to which the framework was valuable in this evaluation study. 

This coding will be conducted by one researcher and 10–20% will be independently coded by another researcher to cross-check appropriateness of coding. Throughout the coding process, these two researchers will meet regularly to discuss the codes and applicability of the framework and subsequently derived templates for analyses. Any disagreements will be resolved by discussion with a third researcher.

The quantitative data will be summarised and analysed using descriptive statistics to further understand the acceptability of the intervention proposed in the main study. This data will likewise be modelled to the implementation acceptability framework. This quantitative data will help further interpret results from the qualitative data, highlighted triangulated key factors which may have varied the level of acceptability.

### Data and safety monitoring

Field workers will record any adverse events and ensure the safety of participants. Field workers will visit all infants daily during the supplementation period to administer the drops as described above and to check on the infant’s health status. If an infant is found unwell, the study nurse will check on the infant and decide on treatment/referral to the MRCG@LSHTM Keneba health centre in consultation with the Research Clinician. Parents/guardians will complete a weekly questionnaire asking whether they have offered foods or fluids other than breast milk.

The trial will be overseen by a DSMB. Safety data will be reviewed by the DSMB as overall pooled data (open session) and tabulated by coded treatment arm (closed session) at given time intervals. Unblinding of the treatment will only be requested by the DSMB in individual cases if there is a pattern of serious adverse events which may be related to study treatment where the DSMB feels there is ‘potential for harm’. Monitoring the rate of recruitment and level of retention of infants and to examine any trends apparent related to non-retention (e.g., consent withdrawal, loss to follow-up etc.) and to provide advice accordingly.

Recruitment to the trial may be paused upon discretion of the DSMB if any infant has a serious adverse reaction to any of the interventions administered during the trial.

In addition to the DSMB, the Local Safety Monitor will regularly review all adverse events and serious adverse events. This review will focus particularly on adverse events causality and reasons for losses to follow up, raising any concerns or issues that present immediate safety concern with the principal investigators for reporting to the DSMB, while protecting the confidentiality of the trial data and the results of monitoring.

Experimental supplements shall be withheld for seven days following detection of any of the following conditions: confirmed fever (axillary temperature >37.5°C) not associated with teething or vaccination ^a^; visually confirmed bloody diarrhea; hospitalization for somatic infection; treatment with antibiotics for any confirmed or suspected somatic infection ^b^.

### Withdrawal of participants

In case the participant decides to withdraw participation or consent during the study, we will not work on the participant’s samples without permission, but any information already generated from the samples until the time of withdrawal will be used and samples already collected, for which they have given consent, will also be analysed and data used. The study clinician may also ask for tests for the participant’s safety. The principal investigator/sub-investigator will ask about the reason for any withdrawal and follow-up with the participant regarding any unresolved adverse events.

### Participant confidentiality

Any identifiable data collected will be stored securely and their confidentiality protected in accordance with the UK General Data Protection Regulation (UK GDPR) and the Data Protection Act (2018). Participant confidentiality, privacy and anonymity will always be ensured. All data will be anonymised, and individuals will not be identifiable.

### Future use of stored specimens and data

Aliquots of blood and faecal samples will be kept frozen at -80°C for future analysis. This may include export of samples. We will obtain informed consent from the parents/guardians for this within informed consent. The blood and faecal samples collected during the trial may be used to support other research in the future, and may be shared anonymously with other researchers, for their ethically approved projects. Any future use of data or samples will require approvals from the principal investigator, MRCG@LSHTM Scientific Coordinating Committee, and Ethics Committee.

## Ethics

These studies will be conducted in accordance with the principles set forth in the ICH Harmonised Tripartite Guideline for Good Clinical Practice and the Declaration of Helsinki in its current version whichever affords the greater protection to the participants. The studies have been reviewed by the MRCG@LSHTM Scientific Coordinating Committee, The Gambian Government/MRC Unit The Gambia Joint Ethics Committee and the Ethics Committee at LSHTM (Approval IDs: 19092; 21257). All amendments will go through the same process and the changes will be communicated with investigators, trial participants, trial registries, journals, and regulators.

## Funding

Funding for the trial is from DFID/NIHR/MRC/Wellcome Joint Global Health Trials - Call 9. All funds will be used in The Gambia. The study will benefit from the infrastructure already in place at MRCG@LSHTM which are funded by the UK Medical Research Council (MRC) and the Department for International Development (DFID) under the MRC/DFID Concordat agreement.

## Dissemination and data access

All key findings from this study will be submitted for publication in peer-reviewed journals. Our planned dissemination avenues include at least three publications in high impact peer-reviewed open-access scientific journals with a wide readership, presentation at international conferences (e.g., the Micronutrient Forum) and dissemination of the trial findings to international and Gambian organizations.

Any request for use of study data will go through approval from the Sponsor and the Ethics Committee. All data will be in an anonymous format for external users. Data sharing will agree with the Sponsor policy on research data sharing.

## Study status

The pilot study has recruited all 100 infants (November 2021) with the final infant due for follow up in March 2022. The qualitative study focus group discussions have been completed, with the interviews commencing in late December 2021.

## Discussion and conclusion

In infants under six months of age, with normal birth weight, exclusive breastfeeding and delayed cord clamping are the only practices recommended to prevent anaemia by the WHO guidelines ^23^. For low-birth-weight infants, WHO guidelines recommend an external source of iron before six months of age. Maternal iron status is important during pregnancy as iron stores for the first few months of life are passed from mother to infant in utero ^24^. The iron received in utero is used for growth and development in the first six months of life, acting as a buffer for the nutritional needs of new tissue. Randomised trials have shown that antenatal iron supplementation and delayed cord clamping each increase iron stores in neonates ^25^.

This study aims to determine if early supplementation of infants with iron will improve serum iron concentrations without additional adverse effects and without undermining exclusive breastfeeding. This is the first time such an intervention will be done in this age group for this population. Further, the planned mixed methods research will explore the effects of this clinical intervention on relevant outcomes while observing and gathering information on implementation. The use of qualitative and quantitative methods will shed light on acceptability of iron supplementation in young infants through the perspective of local stakeholders and mothers as the primary caregivers.

### Evaluation of risks and benefits

There has been a long-standing controversy regarding iron and infections after the Pemba Trial indicated an increase in malaria-related hospital admissions ^26^. Malaria rates in West and East Kiang are now low. Although there is limited data on young infants in the Gambia, malaria prevalence is low ^27^ and recent data from trials by our group in The Gambia has demonstrated that children in Gambia are not put at risk by receiving iron (personal communication with D.I.A. Pereira; IHAT-GUT) ^9^. Nonetheless, all infants in this trial will be visited daily, both to ensure compliance with taking the iron or placebo and to ensure their safety.

A 2020 systematic review of multiple micronutrients and/or iron supplementation in infants under six months of age concluded that infants less than six months of age benefit biochemically from early supplementation with iron, but the impacts on growth, morbidity and/or mortality, and neuro-behavioural outcomes remain unclear ^28^. Well-powered randomised controlled trials are required to determine whether routine supplementation with iron or multiple micronutrients containing iron should commence before six months of life in exclusively breastfed infants in low-resource settings ^28^. Furthermore, a large portion of published and registered on-going trials have initiated supplements after six months of age; by which time children living in poor communities in rural Africa and elsewhere are already likely to be iron deficient.

In conclusion, before testing the impact of iron supplementation in early infancy in a larger scale trial it is important to run this proof-of-concept trial aiming to show a reverse in decline in serum iron without undermining breast feeding in this setting. Additionally, the mixed methods study will be crucial to help understand the acceptability of this approach. If supplementation works well in this group, it will provide the background information for a larger multi-center, multi-country randomised control trial with behavioural and developmental outcomes.

## Data availability

### Underlying data

No data is associated with this article.

### Extended data

Figshare: Extended Data: Iron supplementation of breastfed Gambian infants from 6 weeks to 6 months of age: Protocol for a randomised controlled trial. https://doi.org/10.6084/m9.figshare.17206319.v1 ^29^.

This project contains the following extended data:

- Participant information sheet and consent form
- R code for randomization

### Reporting guidelines

Figshare: SPIRIT Checklist for ‘Iron supplementation of breastfed Gambian infants from 6 weeks to 6 months of age: Protocol for a randomised controlled trial’. https://doi.org/10.6084/m9.figshare.17143019.v2 ^30^.

Data are available under the terms of the Creative Commons Attribution 4.0 International license (CC-BY 4.0).

## Footnotes

^a^ This includes malaria (confirmed fever plus dipstick-test confirmed parasitemia). In case of 48-hour history of fever as reported by the mother (or guardian) that is not confirmed axillary temperature ≤37.5°C), ask the parent/guardian to bring the infant after six hours, and repeat measurement.

^b^ Suspected ear infections or urinary tract infections in children aged less than six months shall be treated with antibiotics (check with physician) - thus, iron shall be withheld in these cases. If antibiotics are given for localized infections and there is no confirmed fever or a reported 48-hour history of fever, then supplemental iron can be given.
