# Supplementary material for: Iron supplementation of breastfed Gambian infants from 6 weeks to 6 months of age: protocol for a randomised controlled trial
Source: Wellcome Open Res. 2022 Jan 18;7:16. [Version 1] doi: 10.12688/wellcomeopenres.17507.1 (PMC9975413; doi:10.12688/wellcomeopenres.17507.1)
Supplement: Peer review history

# Peer review: Reviewer response for version 1

**DOI:** 10.21956/wellcomeopenres.19357.r53882

**Published:** 2023-01-19

**Authors:** Vicky Fallon

**Affiliations:** 1 Institute of Population Health, University of Liverpool, Liverpool, UK

This is a very well written protocol with excellent clarity on design and methods. The background provides a very clear rationale is well substantiated with recent and appropriate evidence. My only (minor) comment is that it would be useful to use the term 'exclusively breastfed infants' throughout as it was not until the methods section that I understood this to be the case.

Is the study design appropriate for the research question?

Yes

Is the rationale for, and objectives of, the study clearly described?

Yes

Are sufficient details of the methods provided to allow replication by others?

Yes

Are the datasets clearly presented in a useable and accessible format?

Not applicable

Reviewer Expertise:

Reproductive psychology

I confirm that I have read this submission and believe that I have an appropriate level of expertise to confirm that it is of an acceptable scientific standard.

# Peer review: Reviewer response for version 1

**DOI:** 10.21956/wellcomeopenres.19357.r50161

**Published:** 2022-05-18

**Authors:** Tor A. Strand

**Affiliations:** 1 Innlandet Hospital Trust, Department of Research, Norway and the Centre for International Health, University of Bergen, Bergen, Norway

This is a well-written protocol on daily iron supplementation in young Gambian infants. The primary outcome is serum iron concentration. Other more commonly used markers of iron status such as ferritin, transferrin receptor, or iron-binding capacity will also be measured.  

 The introduction builds a good case for the research questions, which are reflected in the main objective; to measure the effect of iron supplementation until six months of age on serum iron concentration. 

 The secondary objectives (effects of iron supplementation on) exclusive breastfeeding, growth, and gut functions lack appropriate justification in the introduction. Moreover, these outcomes are not mentioned when the secondary outcomes are listed later in the manuscript. I suggest that the authors align their description of the secondary outcomes and secondary objectives. 

 The qualitative objectives make sense as the project's purpose is to prepare for a more extensive iron supplementation trial with clinical outcomes. 

**Specifics:**

**Consenting:**

- A significant proportion of the mothers will be illiterate, and a witness will sign. Will there be a registry of the witnesses?

**Baseline:**

- Please explain what is meant by a "cold box" or, later in the manuscript, "cool box" (maybe the authors should use the same term).

**Randomization and blinding: **

- The description of the randomization process, allocation concealment, and blinding is unclear and unnecessarily lengthy. To me, it seems as if two different procedures are described. Please sharpen this part. 

**Laboratory parameters: **

- The hematology and biochemical assays are well described. However, the method to measure serum iron (the primary outcome) is not mentioned. Please do. 
- All the biochemical parameters make sense, but I suggest that the authors should offer a couple of sentences to justify these parameters. I assume that hematology (I.e., analyses of whole blood) is quite resource intensive (full blood that needs to be analysed within a relatively short time).
- Justify and explain the method of analyzing faecal iron. 

**Outcomes:**

- It makes sense that Hb, anemia, and iron status parameters are included as 2nd outcomes. However, they are not mentioned as 2nd objective.
- The iron status parameters will be used as categorical variables; I think that it makes more sense to include them as continuous variables, particularly in this study. Please justify the analytical approach (of using categorical outcome variables).

**Data collection:**

- Four types of data are mentioned, but laboratory data is not included in this description. 
- Missing data, which variables do you consider to impute? And why?

Is the study design appropriate for the research question?

Yes

Is the rationale for, and objectives of, the study clearly described?

Yes

Are sufficient details of the methods provided to allow replication by others?

Yes

Are the datasets clearly presented in a useable and accessible format?

Not applicable

Reviewer Expertise:

Epidemiology, clinical trials, micronutrition, child health, development.

I confirm that I have read this submission and believe that I have an appropriate level of expertise to confirm that it is of an acceptable scientific standard.
